# Investigation of the effects of peanut ball during labor: An updated systematic review and meta-analysis

**DOI:** 10.18332/ejm/201345

**Published:** 2025-03-14

**Authors:** Somayeh Makvandi, Leila Karimi, Mona Larki, Amirhossein Sahebkar

**Affiliations:** 1 Menopause Andropause Research Center, Ahvaz Jundishapur University of Medical Sciences, Ahvaz, Iran; 2 Nursing Research Center, Clinical Research Institute, Faculty of Nursing, Baqiyatallah University of Medical Sciences, Tehran, Iran; 3 Nursing and Midwifery Care Research Center, Mashhad University of Medical Sciences, Mashhad, Iran; 4 Department of Midwifery, School of Nursing and Midwifery, Mashhad University of Medical Sciences, Mashhad, Iran; 5 Center for Global Health Research, Saveetha Medical College and Hospitals, Saveetha Institute of Medical and Technical Sciences, Saveetha University, Chennai, India; 6 Biotechnology Research Center, Pharmaceutical Technology Institute, Mashhad University of Medical Sciences, Mashhad, Iran; 7 Applied Biomedical Research Center, Mashhad University of Medical Sciences, Mashhad, Iran

**Keywords:** childbirth, peanut ball, birth ball, meta-analysis

## Abstract

**INTRODUCTION:**

Peanut balls, a specific type of positioning aid used during labor, have gained attention for their potential to enhance maternal comfort and facilitate fetal positioning. This meta-analysis aims to evaluate the effect of peanut balls on the duration of the first stage of labor, the rate of cesarean section, and maternal satisfaction.

**METHODS:**

A comprehensive literature search was carried out employing electronic databases such as PubMed, Web of Science, and the Cochrane Library. The search included articles published from inception to 11 October 2023 with no language restrictions. Randomized controlled trials or quasi-experimental studies were considered for inclusion if they met the following criteria: participants were pregnant women in labor; intervention involved using a peanut ball during labor; and primary outcome included duration of labor, and the rate of cesarean section and maternal satisfaction were secondary outcomes. The risk of bias in the included studies was assessed using the Risk of Bias 2 (RoB2) tool. Results were synthesized using Review Manager software (RevMan version 5.1), employing both fixed-effect and random-effects models as appropriate, and results were presented as risk ratios for dichotomous outcomes and mean differences and standardized mean differences for continuous outcomes. The quality of the evidence was assessed using GRADEpro GDT (Guideline Development Tool).

**RESULTS:**

Eight studies including 1352 laboring women met the criteria to be included in the systematic review and meta-analysis. The results of the meta-analysis showed that the women with epidural analgesia who used the peanut ball, experienced a shortened duration of the first stage of labor by 52.98 min, which was statistically significant (p=0.003). Heterogeneity evidence was not found among the included studies (χ^2^=6.83, p=0.15, I2=41%). It was also shown that the risk ratio of cesarean section in women who used peanut balls during childbirth was significantly lower than the control group (RR=0.74; 95% CI: 0.60–0.91, p=0.0004) (χ^2^=5.72, p=0.45, I2=0%). Compared to the control group, the women in the peanut ball group were found to have a higher satisfaction level, which was statistically significant (p<0.0001).

**CONCLUSIONS:**

The peanut birth ball reduces the first stage of labor duration, and lowers cesarean rates in women with epidural analgesia. While effective and non-invasive, the findings are limited by the risk of bias in some included studies.

## INTRODUCTION

The labor and birth experience is significant for women and their families, with both immediate and enduring effects^1^. The primary goal of effective labor and birthing management is to promote maternal and neonatal health, safety, and overall well-being, while also ensuring a positive childbirth experience. This includes avoiding unnecessary interventions, reducing anxiety, and optimizing resource utilization^2^. The World Health Organization (WHO) emphasizes the importance of not only ensuring the survival of mothers and infants but also prioritizing their holistic well-being and positive experiences during childbirth^3^.

During the process of childbirth, it is essential to prioritize the women’s autonomy by providing comprehensive information, addressing inquiries, and respecting their preferences. Healthcare teams should aim to minimize interventions to support the natural progression of labor^4^. Non-pharmacological methods that promote a sense of safety, respect, and support can lead to better childbirth experiences^5^. One innovative non-pharmacological technique that has gained attention is the use of peanut balls to enhance labor and birth outcomes in women^6^. While peanut balls have become popular among midwives and birthing experts, research supporting their effectiveness is limited. The guidelines provided by the National Institute for Health and Excellence (NICE), do not explicitly mention the use of peanut balls in labor management^6^.

A 2019 meta-analysis by Grenvik et al.^7^ with four included studies^8-11^ found that total length of labor was 79 min shorter in the peanut ball group compared to the control, but this was not significant. It should be noted that this finding was obtained by analyzing only one randomized controlled trial (RCT)^9^. Nevertheless, there was a contradiction with a systematic review conducted in 2021 by Ahmadpour et al.^12^, which analyzed data from six studies^8-11,13,14^ and concluded that there was no statistically significant difference in the duration of the first stage of labor and the frequencies of cesarean sections. Delgado et al.^15^ performed a systematic review and meta-analysis in 2022 focusing on the application of the peanut ball among laboring women receiving epidural analgesia. The results indicated that the use of the peanut ball, in contrast to its absence, was associated with a notable decrease in the length of the first stage of labor. It is important to highlight that this conclusion was derived from the analysis of only two articles.^8,10^. This study revealed a statistically significant 11% increase in the probability of vaginal birth among participants assigned to the peanut ball intervention group in comparison to those in the control group. There was no significant variation observed in the incidence of cesarean sections or operating vaginal delivery, and there is no evidence of other maternal outcomes^15^.

It seems that conducting a new meta-analysis regarding the effect of peanut ball on laboring women is necessary because the available research presents conflicting findings and the last search was conducted until December 2020. Also, in the current study, in addition to the variables related to childbirth, we evaluated the maternal satisfaction with childbirth as a side finding, which was not considered in any of the meta-analyses mentioned above. It seems that due to the relatively small sample sizes used in the trials, the body of evidence is limited, necessitating a cautious approach when evaluating the findings. Given the significance of addressing robust evidence in providing optimal care to women during childbirth, a comprehensive examination and synthesis of existing literature was performed through a systematic review and meta-analysis to investigate the impact of peanut ball on labor duration (primary outcome), and cesarean section rate and maternal satisfaction (secondary outcomes).

## METHODS

### Information sources and search strategy

A comprehensive search of the literature was conducted using electronic databases including PubMed, Web of Science, and Cochrane Library. The search included articles published from inception to 11 October 2023, with no language restrictions. The following keywords were used in various combinations: ‘peanut ball’, ‘labor’, ‘labour’, ‘delivery’, and ‘childbirth’. Additional relevant studies were identified through manual searches of reference lists from relevant articles and review papers. The search strategies in the different databases are given in the Supplementary file.

### Eligibility criteria

Studies were considered for inclusion if they met the following criteria: 1) randomized controlled trials or quasi-experimental studies; 2) participants were pregnant women in labor with or without epidural analgesia; 3) intervention involved the use of a peanut ball during labor; and 4) primary outcome included duration of labor, and secondary outcomes were the rate of cesarean section and maternal satisfaction. Studies were excluded if they were not original research articles, were letters, qualitative research, or a thesis.

### Study selection

We followed a structured process for study selection. Two reviewers (LK and ML) independently screened the titles and abstracts of all retrieved records to identify potentially eligible studies. Full-text articles of relevant studies were then reviewed independently by the same two reviewers to determine final eligibility based on the inclusion criteria. In case of disagreement, a third reviewer (SM) was consulted to reach a consensus. No automation tools were used in the study selection process.

### Data extraction and data items

Two independent reviewers (SM and ML) utilized a standardized data extraction form to gather information from the studies included in the analysis. The data extracted encompassed various study characteristics, such as the author, year of publication, and country, as well as participant characteristics, including sample size, parity, and inclusion criteria. Additionally, details regarding the intervention, such as the type of peanut ball and duration of use, were recorded, along with outcomes related to obstetric results, including labor duration and delivery mode. Any discrepancies that arose were addressed through discussion and consensus; if necessary, a third reviewer (AS) was consulted to ensure the accuracy of the data.

In terms of data items, we established clear definitions for variables based on the PICO framework:

- Population: Women in labor.
- Intervention: Employment of the peanut ball during labor.
- Comparison: Standard care without the use of the peanut ball.
- Outcomes: Primary outcomes consisted of labor duration, while secondary outcomes included delivery mode and maternal satisfaction.

We aimed to collect all results relevant to each outcome domain across all available measures, time points, and analyses. In instances where multiple measures for the same outcome were reported, we prioritized those that appeared most frequently across the studies. In cases of missing or ambiguous data, we made assumptions only when warranted by other information within the study, and these assumptions were thoroughly documented.

### Risk of bias

The methodological quality of the included studies was assessed using version 2 of the risk-of-bias tool for randomized trials (RoB2) as outlined in the Cochrane Handbook^16^. This tool evaluates five domains where bias might be introduced, including: 1) the randomization process, 2) deviations from the intended interventions, 3) missing outcome data, 4) measurement of the outcome, and 5) selection of the reported result. Each domain, as well as the overall risk of bias for each study, was categorized as either low risk, some concerns, or high risk of bias. Where necessary, additional data were obtained from the authors of the publications. The assessments were conducted independently by two reviewers (SM and ML) and disagreements between researchers were resolved through discussion, with the involvement of a third investigator (AS) when required.

### Grading of evidence

According to the Grading of Recommendations Assessment, Development, and Evaluation (GRADE) criteria^17^, two authors assessed the level of evidence (SM, AS) for the outcomes to judge the reliability of the results. For randomized trials, the assessment was divided into five panels: risk of bias, inconsistency, indirectness, imprecision, and publication bias. The level of evidence quality was classified into four grades: high, moderate, low, and very low.

### Data synthesis and analysis

The analysis was conducted utilizing Review Manager software (RevMan version 5.1), establishing a significance threshold at p<0.05. For dichotomous outcomes, risk ratios (RR) accompanied by 95% confidence intervals (CI) were computed. In the case of continuous outcomes, mean differences (MD) or standardized mean differences (SMD) along with 95% CI were determined.

The assessment of heterogeneity among studies was carried out using the I² statistic, where values exceeding 50% indicated considerable heterogeneity. In instances of significant heterogeneity, a random-effects model was employed; conversely, a fixed-effects model was utilized when heterogeneity was not substantial. Missing summary statistics were addressed through imputation methods when feasible, including the estimation of standard deviations from confidence intervals, p-values, or other available metrics. No further data conversions were executed.

Forest plots were employed to encapsulate information regarding individual studies, offering a visual depiction of study heterogeneity and illustrating the estimated overall effect size. Publication bias was not evaluated due to the limited number of studies represented in each forest plot. Subgroup analyses were performed to investigate potential sources of heterogeneity, such as characteristics of the population. A sensitivity analysis was conducted by excluding studies with a high risk of bias or by applying alternative statistical models to confirm the robustness of the meta-analysis findings.

### Ethical considerations

As this study involved the analysis of published data, ethical approval was not required.

## RESULTS

### Study selection

**Figure 1:**
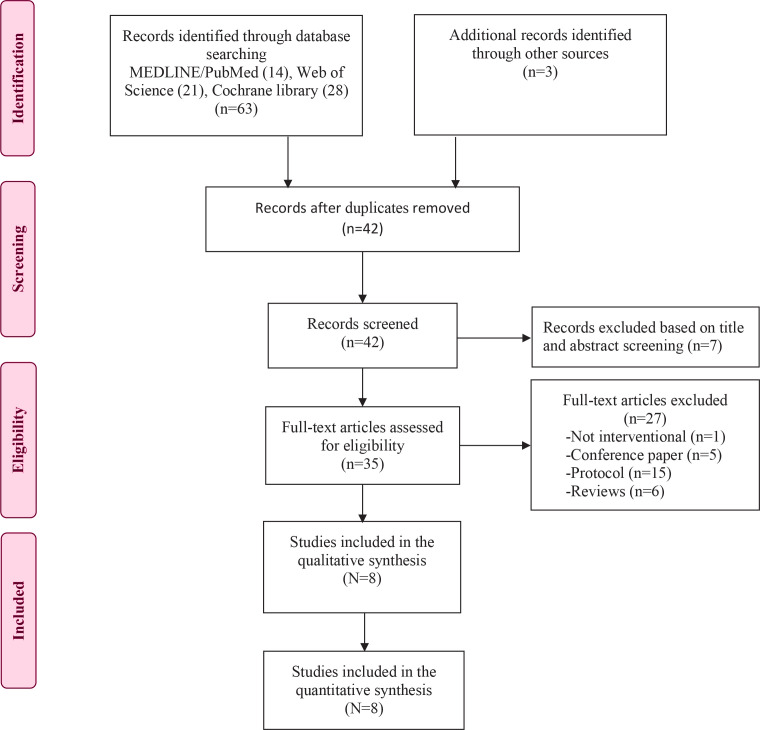
PRISMA flow diagram of studies selection

The findings are reported according to the Preferred Reporting Items for Systematic Reviews and Meta-Analyses (PRISMA) 2020 guidelines. Figure 1 presents the PRISMA flow diagram of study selection. A total of 63 records were gathered from three main databases, along with one record sourced from Google Scholar. Additionally, two studies were selected from the **clinicaltrials.gov** website as they were completed and their results were accessible^11,13^. After removing duplicates using EndNote 20, 42 articles remained. Following a review of titles and abstracts, 7 unrelated articles were removed, leaving 35 articles for the second stage of screening. Finally, after a detailed review of the full texts, 8 studies were deemed eligible for inclusion in the systematic review and meta-analysis.

### Study characteristics

Table 1 shows a summary of the characteristics of the studies included in the systematic review. Six studies were randomized clinical trials^8-11,13,18^, and two were semi-experimental^19,20^. A total of 1352 pregnant women participated in the eight included studies. The number of participants varied from 80^19^ to 382^20^ per study. In five studies, nulliparous women^8,11,13,18,19^ and in three remaining studies^9,10,20^, a combination of nulliparous and multiparous women was included. In six studies, pregnant women who received epidural anesthesia during labor^8-11,13,20^, in the other two studies, women without epidural anesthesia were included^18,19^. Six studies were conducted in the United States^8-11,13,20^ and one each in Turkey^18^ and Egypt^19^, including articles published between 2014^11^ and 2023^18^.

**Table 1:** Summary of characteristics of studies included in the systematic review

*Authors* *Year* *Country*	*Study design*	*Participants*	*IG*	*CG*	*PB size*	*Outcomes*	*Findings*
Ahmed et al.^19^ 2022 Egypt	Semi-experimental	80 low-risk nulliparous 20–35 years 37–42 weeks of gestation, singleton, in the active phase of 1st stage of labor	n=40 PB exercises during the active phase of first stage of labor. Placed between the legs of a woman at the early active phase of the first stage and assisted with turning or changing position and adjusting the peanut ball every 1 hour, the positions used were left lateral, right lateral or semi-Fowler. It remained in place until the cervix was completely dilated.	n=40 routine hospital care	45×85 cm	Duration of labor Mode of delivery	Shorter duration of the first and second stage in the IG compared to the CG less CS in the IG compared to the CG
D’Angelo^13^ 2015 US	RCT	81 nulliparous 18–50 years ≥37 weeks gestation, not allergic to medications used for labor analgesia	n=39 PB after labor analgesia will be utilized until complete cervical dilation (no additional data)	n=42 no intervention	no data	Duration of labor Mode of delivery Maternal satisfaction	Shorter duration of the labor and more satisfaction in the IG compared to the CG less CS in the IG compared to the CG
Evans and Cremering^11^ 2014 US	RCT	191 nulliparous women age ≥18 years, presenting in labor, gestational age of 37 to 42 weeks, single gestation, vertex presentation, plans to deliver with epidural anesthesia	n=91 Use of the PB within 30 minutes after epidural placement (no additional data)	n=100 standard care for positioning during labor using pillows and wedges	no data	Duration of labor Mode of delivery 3rd and 4th degree lacerations	Longer duration of the first and second stage in the IG compared to the CG less CS in the IG (23%) compared to the CG (31%) lower rate of the 3rd and 4th degree lacerations on IG compared to the CG
Hickey and Savage^20^ 2019 US	Semi-experimental	382 nulliparous and multiparous >18 years, low risk, received epidural anesthesia, at least 37 weeks gestation, singleton fetus in vertex presentation	n=203 PB was placed after epidural administration; women were repositioned a minimum of every 1 to 2 hours, and the PB was removed at second stage of labor. Positions used were side-lying, tuck, and semi sitting lunge	n=179 standard care	appropriately sized PB (no data)	Duration of labor Mode of delivery	No differences in duration of the first stage of labor in the IG compared to the CG Women in the PB group were 50% less likely to have CS
Mercier and Kwan^8^ 2018 US	RCT	86 low-risk nulliparous >18 years, received epidural anesthesia, singleton, cephalic presentation, gestational age of 37 to 41.6 weeks	n=43 The women were required to use the PB (between knees) for a minimum of 15 minutes of each hour of subsequent labor, though more frequent use was allowed if the patient preferred	n=43 standard nursing care practices	no data	Rate of cervical dilation Duration of labor Mode of delivery	No statistically significant difference in rates of cervical dilation, duration of labor, and mode of delivery between the groups
Roth et al.^9^ 2016 US	RCT	149 nulliparous and multiparous 18–35 years, received epidural anesthesia, gestational age of ≤39 weeks	n=78 Women had the PB placed between their knees within 30 minutes after epidural placement, with rotation of lateral positions every 30 minutes or as indicated by patient/fetal status.	n=71 used a maximum of one pillow between the knees	no data	Labor duration	Shorter duration of the first stage of labor for primiparous No decrease in first stage labor duration was shown for multiparous patients and no significant decrease in pushing time was shown for either primiparous or multiparous patients
Sönmez and Ejder Apay^18^ 2023 Turkey	RCT	180 nulliparous 38–40 weeks of gestation, estimated fetal weight of <4000 g, normal pelvic diameter, singleton pregnancy, 4 cm of cervical dilation	IG1(60): spherical BB exercises IG2(60): PB exercises included five different positions (half-sitting position, tucked side-lying position, hands and knee fire hydrant position, straddling position, forward-leaning position) and movements suitable to these positions (jumping, right-left, front-back on the ball)	n=60 routine midwifery care	based on the height	Labor pain Satisfaction Rate of fetal head descent	Lower labor pain, higher rate of fetal head descent, and higher maternal satisfaction at the first stage of labor in IGs compared to the CG
Tussey et al.^10^ 2015 US	RCT	203 nulliparous and multiparous 18–35 years, received epidural anesthesia, gestational term pregnancy	n=107 The PB was placed between the legs of a woman immediately after she received her epidural. It was removed when the cervix of the woman became completely effaced and dilated, passive descent had occurred.	n=94 standard care	no data	Duration of labor Mode of delivery	Shorter duration of the active phase and second stage in the IG compared to the CG Less CS in the IG compared to the CG

PB: peanut ball. BB: birth ball. RCT: randomized controlled trial. IG: intervention group. CG: control group. CS: cesarean section.

### Risk of bias description

The risk of bias for the eight studies is shown in Table 2. The randomization process in two studies was rated as high risk^19,20^, and in six studies it used allocation concealment and was therefore rated as low risk^8-11,13,18^. The domain of deviations from intended interventions was rated as some concern and the domains of missing outcome data, measurement of the outcome, and selection of the reported result were rated as low risk in all of the studies. Consequently, the overall bias was high risk in two studies^19,20^ and some concern in six studies.

**Table 2:** Risk of bias judgment for included trials

*Authors* *Year*	*D1*	*D2*	*D3*	*D4*	*D5*	*Overall*
Ahmed et al.^19^ 2022	High concerns	Some concerns	Low	Low	Low	High risk of bias
D’Angelo^13^ 2015	Low	Some concerns	Low	Low	Low	Some concerns
Evans and Cremering^11^ 2014	Low	Some concerns	Low	Low	Low	Some concerns
Hickey and Savage^20^ 2019	High concerns	Some concerns	Low	Low	Low	High risk of bias
Mercier and Kwan^8^ 2018	Low	Some concerns	Low	Low	Low	Some concerns
Roth et al.^9^ 2016	Low	Some concerns	Low	Low	Low	Some concerns
Sönmez and Ejder Apay^18^ 2023	Low	Some concerns	Low	Low	Low	Some concerns
Tussey et al.^10^ 2015	Low	Some concerns	Low	Low	Low	Some concerns

D1: bias arising from the randomization process. D2: bias due to deviations from intended interventions. D3: bias due to missing outcome data. D4: bias in measurement of the outcome. D5: bias in selection of the reported result.

**Figure 2:**
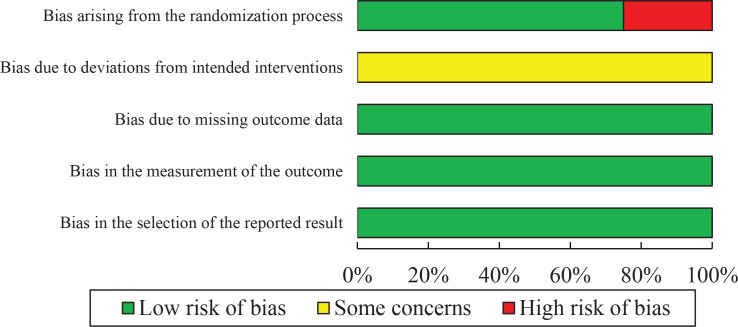
Risk of bias graph across all included studies

Table 2 summarizes the classification of studies according to the five domains of the RoB2 tool. Two studies were rated as high risk in the randomization process^19,20^. Four studies employed low risk randomization and adequate allocation concealment and were rated as low risk in this domain^8-11,13,18^. For the domain of deviations from intended interventions, all studies were rated as having some concerns, primarily due to the absence of blinding of participants and personnel. The remaining domains – missing outcome data, measurement of the outcome, and selection of the reported result – were rated as low risk in all studies. Overall, two studies^19,20^ were classified as high risk of bias, while the remaining six studies were categorized as having some concerns^8-11,13,18^. To provide a cumulative visual assessment, a risk of bias bar chart summarizing the ratings across all studies was created (Figure 2). This chart uses color coding (green for low risk, yellow for some concerns, and red for high risk) to illustrate the proportion of studies falling into each risk category across the five domains.

### Quality of evidence

We used GRADEpro GDT (Guideline Development Tool) to assess the quality of evidence for the two primary outcomes, and the results are summarized in Table 3. The quality of evidence for the duration of the first stage of labor was moderate. This outcome was downgraded by one level due to risk of bias as all included studies had some concerns regarding bias. The intervention showed a significant reduction in the duration of the first stage of labor compared to the control group. While the results suggest a potential benefit of the intervention, the moderate quality of evidence underscores the need for further well-conducted trials to confirm these findings. The quality of evidence for the cesarean section rate was low. This outcome was downgraded by two levels due to risk of bias as two studies^19,20^ had a high risk of bias and the remaining studies had some concerns regarding bias. The low quality of evidence indicates a considerable degree of uncertainty about the effect of the intervention on the cesarean section rate, warranting cautious interpretation of the findings.

**Table 3:** GRADE evidence profiles for three two outcomes among the studies included in the meta-analysis

*Certainty assessment*							*Number of patients*		*Effect*		*Certainty*	*Importance*
*Number of studies*	*Study design*	*Risk of bias*	*Inconsistency*	*Indirectness*	*Imprecision*	*Other considerations*	*peanut ball*	*control*	*Relative (95% CI)*	*Absolute (95% CI)*		
**Duration of the active phase first stage of labor**												
5	randomized trials	serious^a^	not serious	not serious	not serious	none	281	256	-	MD=52.98 lower (87.96 lower to 18 lower)	⨁⨁⨁◯ Moderate	Critical
**Cesarean rate**												
7	randomized trials	Very serious^b^	not serious	not serious	not serious	none	69/305 (22.6%)	89/298 (29.9%)	RR=0.77 (0.59–1.00)	69 fewer per 1000 (from 122 fewer to 0 fewer)	⨁⨁◯◯ Low	Critical

MD: mean difference. RR: risk ratio.

a The overall bias across all studies is classified as ‘some concerns’.

b The overall bias for two studies is ‘high’, while the remaining studies are categorized as ‘some concerns’.

### Meta-analysis findings

*Effect of the peanut ball on the duration of the first stage of labor*

**Figure 3:**
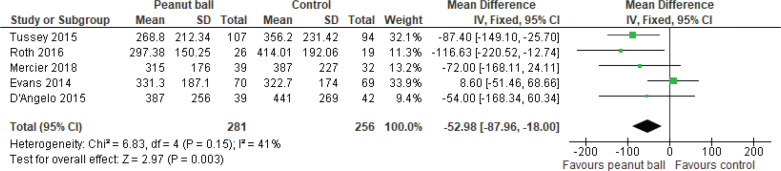
Forest plot of the pooled mean differences of peanut ball effect on the duration of the first stage of labor in women with epidural analgesia (a side-lying position with the ball placed between legs)

Figure 3 shows the forest plot of the peanut ball effect on the duration of the first stage of labor in women with epidural analgesia (a side-lying position with the ball placed between legs). Compared with the control group, the women using peanut balls experienced a shortened duration of the first stage of labor by 52.98 min (95% CI: -87.96 – -18.00), which was statistically significant (p=0.003). Heterogeneity evidence was not found among the included studies, so a fixed-effect model was used (χ^2^=6.83, p=0.15, I^2^=41%). Exclusion of the trials did not impact statistical heterogeneity.

*Sensitivity analysis*

Sensitivity analysis for the duration of the first stage of labor revealed that removing the study by Tussey et al.^10^ caused the findings to shift toward non-significance, with the p-value increasing from 0.003 to 0.9. However, sequential removal of other individual studies did not affect the overall significance, which consistently favored peanut ball use. This highlights the potential influence of the Tussey et al.^10^ study on the robustness of the findings.

*Effect of the peanut ball on the rate of cesarean section*

**Figure 4:**
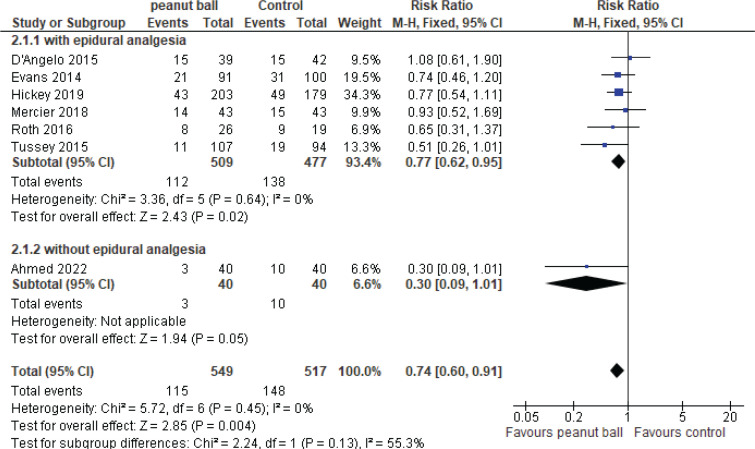
Forest plot of the pooled relative risk of peanut ball effect on cesarean rate in women in a side-lying position with the ball placed between legs

Figure 4 shows the forest plot of the peanut ball effect on cesarean rate in women in a side-lying position with the ball placed between legs. The RR from seven studies^8-10,19,20^ involving 1066 women was 0.74 (95% CI: 0.60– 0.91, p=0.004) based on a fixed-effect model, with non-significant heterogeneity between studies (χ^2^=5.72, p=0.45, I^2^ = 0%). Figure 3 shows the forest plot of individual effect sizes within each study. Analysis of subgroups based on having or not having epidural anesthesia showed that in the group with epidural anesthesia, the RR of cesarean section was significantly lower in the peanut ball group than in the control group (RR=0.77; 95% CI: 0.62–0.95, p=0.02). Evaluation of the publication bias was not possible due to the limited number of studies.

### Sensitivity analysis

The sensitivity analysis concerning the cesarean section rate revealed that the sequential exclusion of individual studies did not alter the overall findings, which consistently demonstrated no significant effect of the intervention. Nevertheless, due to concerns about the validity of meta-analyses based on quasi-experimental studies, we undertook an additional sensitivity analysis by omitting these studies^19,20^. A reanalysis involving 604 women from five clinical trials was conducted, resulting in an effect size that continued to favor the peanut ball group (RR=0.77, 95% CI: 0.59–1.00, p=0.05). This outcome indicates a possible advantage of the intervention, although the borderline significance underscores the necessity for further rigorous clinical trials.

*Effect of the peanut ball on maternal satisfaction*

**Figure 5:**
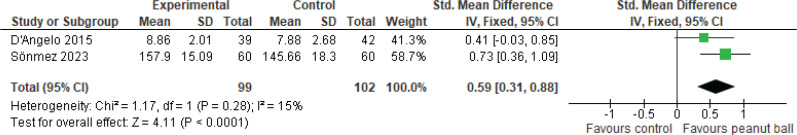
Forest plot of the standard mean differences of peanut ball effect on maternal satisfaction

Figure 5 shows the forest plot of the peanut ball effect on maternal satisfaction. Compared with the control group, the women in the peanut ball group were found to have a higher satisfaction level which was statistically significant (SMD=0.59; 95% CI: 0.31–0.88, p<0.0001). Heterogeneity evidence was not found among the included studies, so a fixed-effect model was used (χ^2^=1.17, p=0.28, I^2^=15%). Sensitivity analysis was not performed because only two studies were available for this outcome. The limited number of studies precluded meaningful sensitivity analyses, as removing one study would leave only a single study, thus making reanalysis impractical and unreliable.

## DISCUSSION

This meta-analysis highlights the significant benefits of using a peanut ball during labor for women. The findings demonstrated a statistically significant reduction in the duration of the first stage of labor, favoring the peanut ball intervention. Similarly, the meta-analysis showed a notable reduction in the cesarean delivery rate associated with the use of the peanut ball. Furthermore, maternal satisfaction was significantly higher among women who used the peanut ball. These findings align with the study objectives, offering robust evidence to support the effectiveness of this intervention. The novelty of this meta-analysis lies in its comprehensive approach to evaluating the impact of the peanut ball on multiple labor outcomes, underscoring its potential as a simple, non-invasive tool to improve childbirth experiences.

The reason for the decrease in the length of the first stage of labor, leading to faster progress, is explained in the study of Ahmed et al.^19^: that the use of the peanut ball significantly increased the number of uterine contractions per 10 min, increased duration, and decreased the interval of uterine contractions among the study group compared to the control group. This increase in uterine contractions’ intensity, duration, and decreased interval, resulted in a significant increase in cervical dilatation and the downward movement of the fetal head. Studies have shown that the fetal pole descends into the pelvic cavity even when the patient is semi-flexed or in a lateral decubitus position. This is because of gravity, which causes the cervix to have more effacement and dilation^21-23^. The mechanism is similar to the effect of birth balls, which accelerate the duration of fetal head descent. A birth ball increases the pelvic outlet by 30%, relaxes connectives, sacroiliac joints, and muscles in the pelvic region, decreases pressure on the bladder, back, and coccyx, increases blood flow to the uterus, optimizes fetal blood circulation, and facilitates rapid descent of the fetus through the influence of gravity^22,24,25^.

The findings of Payton^14^ indicate that the peanut ball simulates a sitting or squatting position during labor by maximizing the diameter of the pelvis, allowing the fetus to have the greatest ability to descend. This promotes the effectiveness of uterine contractions, leading to cervical dilation and improved progress in labor. The peanut ball additionally operates by promoting spinal flexion, which raises the utero–spinal angle. This facilitates moving the occiput posterior to a better position for delivery and speeds up labor^10^. The current finding is in agreement with the study of Sheishaa et al.^26^ which found that the average total Bishop score (including dilation, effacement, etc.) was significantly higher in the peanut birth ball group. However, the study of Payton^14^ demonstrated that using a peanut birth ball alone did not shorten the first stage of labor, and the second stage was significantly longer in the intervention group than in the control group. This difference may be attributed to the non-randomized nature of the study, the inclusion of all women regardless of gravidity, the lack of consistency in the size of the peanut balls, as well as the absence of detailed instructions for staff on their use.

The study of Ahmadpour et al.^12^ showed no significant difference in the rate of cesarean section between the peanut ball group and the control group. Nevertheless, our findings revealed that women who used the peanut ball had a lower rate of cesarean birth. These differences could be attributed to high heterogeneity, insufficient evidence, and a smaller sample size.

According to subgroup analysis, the use of peanut balls in women who received epidural anesthesia reduced cesarean section rates. This can be explained by the fact that the peanut ball, positioned between a woman’s legs in the lateral recumbent position during labor, mimics the upright position, promoting increased maternal mobility, widening the pelvis, improving the efficacy of uterine contractions, and facilitating fetal descent^7^. Delgado et al.^15^ showed that using the peanut ball reduces the duration of the first stage of labor after an epidural and increases the chance of a vaginal birth, although this result differs from our findings.

Some studies reported that participants who used a peanut ball for labor expressed feelings of relaxation and satisfaction, leading to a decrease in stress and tension during labor, facilitating the labor process, and this seems to be another benefit of using a peanut ball in childbirth^18^.

### Strengths and limitations

This meta-analysis has several strengths. Our search strategy was comprehensive and clear, and we did not apply any language or time restrictions. It incorporates all relevant randomized controlled trials available on the subject, and the majority of these studies exhibit a satisfactory level of quality. While this study provides valuable insights into the effects of peanut ball use during labor, several limitations must be acknowledged. The small number of included studies and the potential risk of bias in the evidence may affect the generalizability of the findings. These limitations underscore the need for further well-designed RCTs to confirm these findings and to explore additional clinically relevant outcomes. Another issue is that determining the duration of labor is challenging since it often depends on when a woman initially presents for labor, if labor is spontaneous. If labor is induced, it is still challenging because of the variability in techniques employed for cervical ripening. Nevertheless, the fact that all studies in the analysis of the effect size of the intervention on the duration of the first stage of labor were randomized should mitigate some of this variability.

## CONCLUSIONS

The peanut birth ball appears to significantly reduce the length of the first stage of labor, ultimately contributing to improved labor progress, a lower rate of cesarean delivery in women with epidural analgesia, and a higher rate of maternal satisfaction. This non-pharmacological technique is considered effective, inexpensive, innovative, non-invasive, and easy to learn, especially for women with restricted movement during labor.

While the results of this meta-analysis are statistically significant, the current evidence on the use of the peanut ball during labor is limited, and this type of ball is still unknown in many countries. More systematic reviews and meta-analyses from additional randomized controlled trials with increased sample sizes are needed to accurately determine the true effects of the peanut ball during labor. Based on the current study’s findings, it is suggested that labor wards consider making peanut balls available as an innovative and no-risk non-pharmacologic adjunct to labor management, especially for laboring women who indicate staying in bed during labor. Midwives should be trained to use non-pharmacological interventions, especially the peanut ball. Additionally, further studies with a low risk of bias are recommended for better generalization and validation of the results.

## Supplementary Material



## Data Availability

Data sharing is not applicable to this article as no new data were created.
