# Supplementary material for: Investigation of the effects of peanut ball during labor: An updated systematic review and meta-analysis
Source: Eur J Midwifery. 2025 Mar 14;9:10.18332/ejm/201345. doi: 10.18332/ejm/201345 (PMC11907655; doi:10.18332/ejm/201345)
Supplement: Supplementary file 1 [file EJM-9-15-s1.pdf]

Supplementary Appendix 1

SEARCH STRATEGY

Medline via PUBMED interface:
("Labo*r"[All Fields] OR "childbirth*"[All Fields] OR "parturition*"[All Fields] OR "birth*"[All Fields] OR "pregnan*"[All Fields]) AND "peanut ball*"[All Fields] AND 1985/01/01:2023/12/31[Date - Publication]

Web of Science
(((((((TS=(labo*r*)) OR TS=(birth*)) OR TS=(childbirth*)) OR TS=(delivery)) OR TS=(pregnan*)) OR TS=(Parturition*)) AND TS=("peanut ball*")) AND DOP=(1945/2024)

Cochrane library
Date Run: 11/10/2023 13:47:25

ID	Search	Hits
#1	(birth*):ti,ab,kw OR (lab*r):ti,ab,kw OR (deliver*):ti,ab,kw OR (pregnan*):ti,ab,kw OR (parturition*):ti,ab,kw (Word variations have been searched)	
#2	(peanut NEXT ball*):ti,ab,kw (Word variations have been searched)	
#3	#1 AND #2	

© 2025 Makvandi S. et al.
